# A novel glycyrrhizin acid-coated stent reduces neointimal formation in a rabbit iliac artery model

**DOI:** 10.3389/fphar.2023.1159779

**Published:** 2023-05-17

**Authors:** Shuai Teng, Zhaowei Zhu, Yang Li, Xinqun Hu, Zhenfei Fang, Zhenjiang Liu, Shenghua Zhou

**Affiliations:** ^1^ Department of Cardiovascular Medicine, the Second Xiangya Hospital, Central South University, Changsha, Hunan, China; ^2^ Department of Vascular Surgery, Xiamen Cardiovascular Hospital, Xiamen University, Xiamen, Fujian, China

**Keywords:** drug-eluting stents, glycyrrhizin acid, intimal hyperplasia, eNOS, re-endothelialization

## Abstract

**Introduction:** Most drug-eluting stents (DESs) inhibit intimal hyperplasia but impair re-endothelialization. This study aimed to evaluate in vivo strut coverage and neointimal growth in a new glycyrrhizin acid (GA)-eluting stent.

**Methods:** New Zealand White rabbits (*n* = 20) with atherosclerotic plaques were randomly divided into three groups based on implanted iliac artery stents: bare-metal stents (BMSs), rapamycin-eluting stents, and GA-eluting stents. After the *in vivo* intravascular ultrasound (IVUS) assessment at 28 days, the vessels were harvested for scanning electron microscopy (SEM) and histology. After 4 weeks of follow-up, the stent and external elastic lamina (EEL) areas were compared among the groups.

**Results:** The rapamycin- or GA-eluting stents significantly reduced the neointimal area compared with BMSs, though GA-eluting stents had the lowest reduction. There were more uncovered struts for rapamycin-eluting stents than those for GA-eluting stents and bare-metal stents. The endothelial nitric oxide synthase (eNOS) expression in GA-eluting stents was much higher than that in BMSs and rapamycin-eluting stents, even though the endothelial coverage between struts was equivalent between BMSs and GA-eluting stents. Moreover, GA-eluting stents markedly promoted re-endothelialization and improved arterial healing compared to rapamycin-eluting stents in a rabbit atherosclerotic model.

**Conclusion:** In conclusion, the novel GA-coated stent used in this study inhibited intimal hyperplasia and promoted re-endothelialization.

## 1 Introduction

Coronary artery disease is the most prevalent type of heart disease and the primary cause of mortality in developed and developing countries (Sanchis-Gomar et al., 2016). The increasing use of percutaneous coronary interventions (PCIs) or stents has improved the prognosis of patients with coronary artery disease and acute coronary syndrome (Dudek et al., 2019).

After years of investigation, two important pathological processes have been identified after stent deployment: intimal hyperplasia and re-endothelialization, which are mainly driven by smooth muscles and endothelial cells, respectively (Van Belle et al., 1997). Since smooth muscle cell proliferation is triggered by sterile inflammation and foreign body reactions, intimal hyperplasia clinically leads to lumen loss or in-stent restenosis (Van Belle et al., 1997; Chaabane et al., 2013). However, re-endothelialization is the process of covering the surface of a stent with endothelial cells, thus protecting it from thrombus formation. Restenosis and stent thrombosis are associated with adverse clinical outcomes after stent application (Lu et al., 2016).

Although the application of drug-eluting stents (DESs) has greatly reduced the rate of restenosis compared to bare-metal stents (BMSs), the rate of restenosis remains high (Giustino et al., 2022). Drugs that elute off-current DESs, including sirolimus, paclitaxel, everolimus, and zotarolimus, have strong antiproliferative effects but lack cellular specificity, resulting in delayed re-endothelialization and endothelial dysfunction, which is linked to stent thrombosis (Xu et al., 2022). Owing to inadequate endothelial healing, late stent-associated thrombosis may develop following DES implantation, despite the regular use of extended dual antiplatelet therapy (Finn et al., 2007).

Even in advanced generations, most DESs are not designed with a focus on resolving re-endothelialization and endothelial dysfunction. Our previous study indicated that glycyrrhizin acid (GA) could protect against endothelium-dependent relaxation in an animal model of diabetes (Zhu et al., 2020a) and attenuate neointimal formation by inhibiting HMGB1 in a rabbit vascular injury model (Zhu et al., 2020b). Therefore, this study aimed to investigate the anti-restenotic and anti-inflammatory properties of GA-eluting stents in a rabbit model of atherosclerosis using intravascular ultrasound (IVUS) after stent implantation.

## 2 Materials and methods

### 2.1 Induction and identification of atherosclerosis

All animal experiments were approved by the Animal Care and Use Committee of Central South University. A total of 20 adult male New Zealand White rabbits (3 months old, 3.0–3.5 kg) were purchased from the Shanghai Animal Administration Center (Shanghai, China). For the first 4 weeks of the trial, all rabbits were fed with a high-cholesterol diet (purified rabbit chow supplemented with 1% cholesterol and 6% peanut oil; SLACCAS, Shanghai, China), followed by a low-cholesterol diet (0.025%) for the remaining time (i.e., 4 weeks after stent implantation).

Oil Red O staining was performed to detect vascular atherosclerosis. Vascular sections were rinsed with 60% isopropanol (5 min), stained with 0.5% Oil Red O/60% (20°C, 10 min), destained for 2 min, and thoroughly washed with distilled water. The images were acquired using a microscope (ZEISS).

### 2.2 Construction of the DESs

GA-eluting stents were prepared by coating a cobalt–chromium alloy stent (APT Medical Company, China) with a monolithic matrix of polyvinylidene fluoride-co-hexafluoropropylene and a GA base. The CA-eluting stents were 15 mm long and 2.50 mm in diameter, with a strut thickness of 88 um and a polymer layer thickness of 5–20 µm on the wire. Polyvinylidene fluoride-co-hexafluoropropylene and GA were sequentially dissolved and mixed in acetone to form a coating polymer, and the stents were coated using ultrasonic spraying equipment. Subsequently, the stent underwent a 10-min air-drying process to eliminate acetone completely. Each coated stent contained 100 μg of GA. The integrity and homogeneity of the coated stents were evaluated using a stereomicroscope under white light before and after balloon catheter inflation. This polymer coating does not crack or flake during stent expansion and has been used in other DESs (Kamberi et al., 2018). After the stent was attached to the angioplasty balloon, it was sanitized using the ethylene oxide gas method and aseptically enclosed.

The kinetics of GA elution from polymer-coated scaffolds that had undergone sterilization were assessed *in vitro*. The GA-eluting stent was immersed in phosphate-buffered saline ( pH 7.4 ) in an Eppendorf tube and shaken in a horizontal shaker at 50 rpm, at 37°C. The stents were removed at a set time, a certain amount of acetonitrile was added to the tube and left to stratify, and the supernatant was collected, filtered, and tested by UV spectroscopy at 258 nm. During *in vitro* elution of GA-eluting stents, the GA-eluting stents released 60% of its total GA amount by day 1 and 88% by day 13 (Supplementary Figure S1). Moreover, bare-metal stents (APT Medical Company, China) of the same size and commercially available rapamycin-eluting stents (Partner durable-polymer SES; Lepu Medical Technology, China) were used.

### 2.3 Stent placement, harvest, and preparation

General anesthesia (sodium pentobarbital; 100 mg/kg IV) was administered to the rabbits. A median neck incision was made, and a 5F vascular sheath was placed in the left carotid artery. After the administration of heparin (100 IU/kg), aortography and bilateral iliac angiography were performed. The rabbits (*n* = 20) were randomly divided into three groups as follows: bare-metal stents (BMS group, *n* = 6), rapamycin-eluting stents (rapamycin group, *n* = 6), and GA-eluting stents (GA group, *n* = 8). The operators were unaware of the group assignments, and the stents looked similar. A balloon-to-artery ratio of approximately 1.2:1 was achieved by manually crimping each stent onto a 2.5-mm angioplasty balloon before deploying it in the proximal bilateral iliac artery with 8-atm balloon inflation for 30 s. Aspirin (40 mg) was administered orally to the rabbits 2 days before surgery and thereafter (Nakazawa et al., 2016). Euthanasia was performed 4 weeks after stent deployment, followed by harvesting. The same type of stent was implanted in both iliac arteries of the rabbits, one for scanning electron microscopy (SEM) and the other for histological and immunostaining analyses.

### 2.4 SEM and morphometric analyses

The luminal surfaces of the stented iliac arteries were exposed by longitudinally severing them in half. Frontal SEM images of half of the stent were taken at low power (×15 magnification) to evaluate neointimal development throughout the luminal stent surface visually. The percentage of endothelial coverage was visually evaluated after the images were gradually enlarged (×600).

### 2.5 Histological analysis of neointimal hyperplasia

The stented sections were stained with hematoxylin and eosin. The external elastic lamina (EEL; mm^2^), internal elastic lamina (IEL; mm^2^), and lumen area (LA; mm^2^) were measured using an imaging analysis system (Imagine-Pro Plus). Moreover, the following formulas were used to calculate the neointimal area (NA; mm^2^) and percentage of stenosis: NA = (IEL ˗ LA) and percentage stenosis (%) = [(IEL ˗ LA)/IEL × 100].

### 2.6 Endothelial nitric oxide synthase immunostaining

The longitudinally dissected half of the stent was dissected under a low-power (×10 magnification) microscope to separate the hyperplastic endothelium covering the stent struts. The dissected stent was fixed, paraffin-embedded, and sliced into sections (5 μm thick). After overnight incubation with eNOS antibody markers (1:100; BD Biosciences, CA, United States), the paraffin-embedded slides were incubated with the secondary antibody donkey anti-mouse Alexa Fluor 488 (1:150 dilution; Invitrogen Corp., Carlsbad, California). The nuclei were counterstained with DAPI. The images were captured using an IX73 fluorescence microscope (Olympus, Tokyo, Japan).

### 2.7 *In vivo* evaluation by IVUS after 28 days

IVUS was conducted as described previously (Cui et al., 2017). We used a 40-MHz 2.9 Fr sheath-based catheter (Atlantis, SR Pro, Boston Scientific) and IVUS (iLAB^TM^ Ultrasound Imaging System, Boston Scientific, Natick, MA, United States). The IVUS catheter was positioned 10 mm distal to the stent, and imaging was subsequently performed back to a point 10 mm proximal to the treated section using an automated transducer pullback at 0.5 mm/s. The scaffold area, minimal lumen area, intrascaffold neointimal area, and vessel area (area in the vessel’s EEL) were measured. The stent expansion index and percentage of lumen area stenosis were calculated according to the following formulas: stent expansion index = ([actual lumen area/ideal lumen area] × 100) and percentage of lumen area stenosis = ([mean lumen intrascaffold area ˗ the lumen area]/mean lumen intrascaffold area × 100).

### 2.8 Statistical analyses

All results are expressed as means ± standard error. Statistical analyses were performed using Student’s *t*-test or analysis of variance (ANOVA)/Dunnett’s *t*-test of variance. All analyses were performed using GraphPad Prism 8.0 (GraphPad Software Inc., San Diego, CA, United States of America), and a *p*-value <0.05 (two-sided) was considered to be significant.

## 3 Results

### 3.1 Confirmation of atherosclerosis of the artery

**FIGURE 1:**
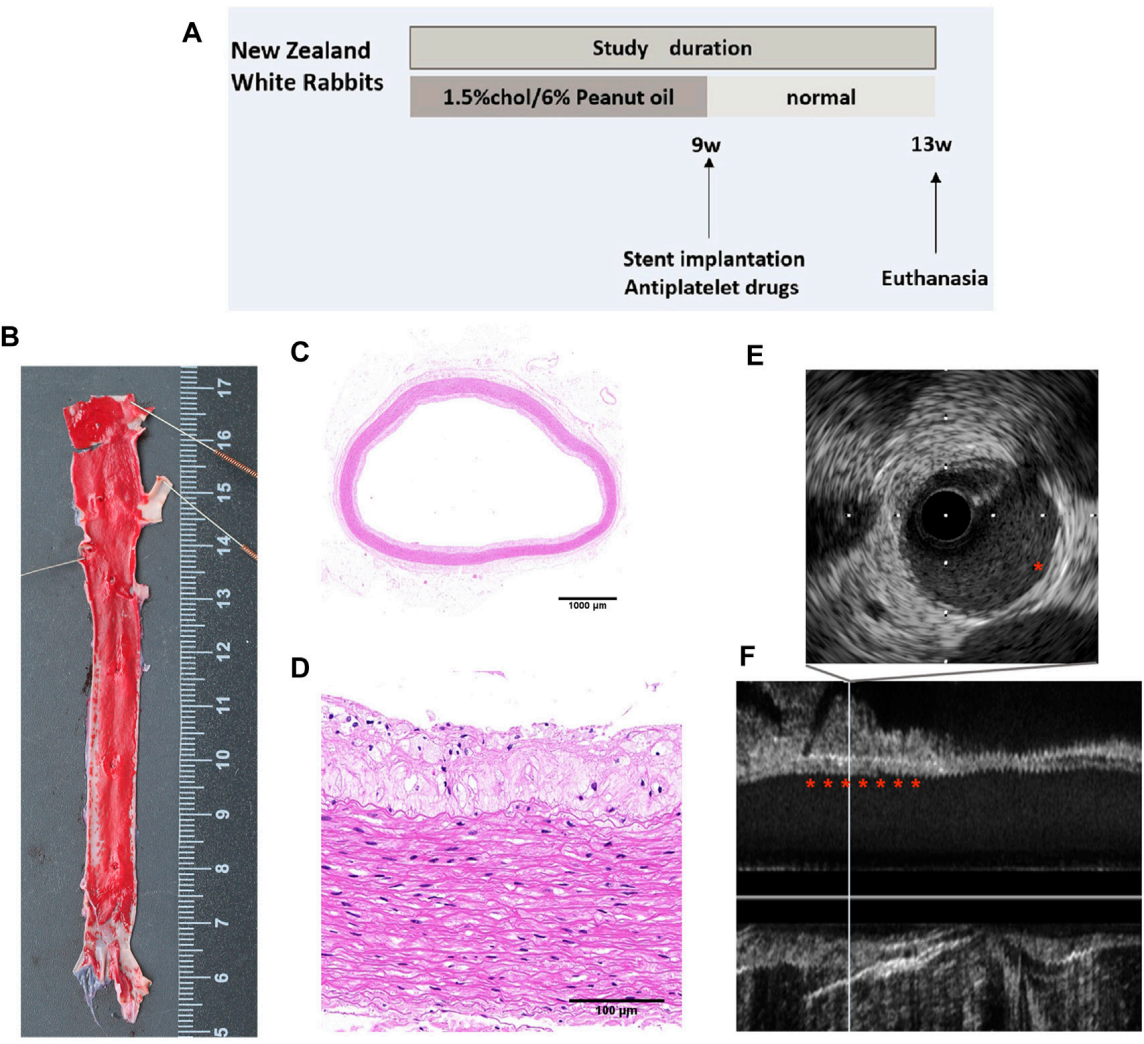
Atherosclerosis model of rabbits. **(A)** Study flow for the induction of atherosclerosis in rabbits. **(B)** Oil Red O staining of the iliofemoral artery. Representative cross-sectional images of H&E-stained iliofemoral arteries at **(C)** low- (×1) and **(D)** higher-power (×20) magnifications. **(E)** Cross-sectional images and **(F)** longitudinal view of IVUS images showing the atherosclerotic aorta.

Figure 1A shows a schematic representation of the experimental setup. Oil Red O staining was performed to confirm the success of the rabbit atherosclerosis model. Following Oil Red O staining, atherosclerotic lesions were grossly observed in the aortas of rabbits (Figure 1B). The lesions were sporadically present throughout the iliac artery, as confirmed by hematoxylin and eosin staining of the aorta cross section (Figures 1C, D). Additionally, *in vivo* IVUS showed a mild plaque burden throughout the iliac artery (Figures 1E, F).

### 3.2 Morphometric measurements and histological observations

**FIGURE 2:**
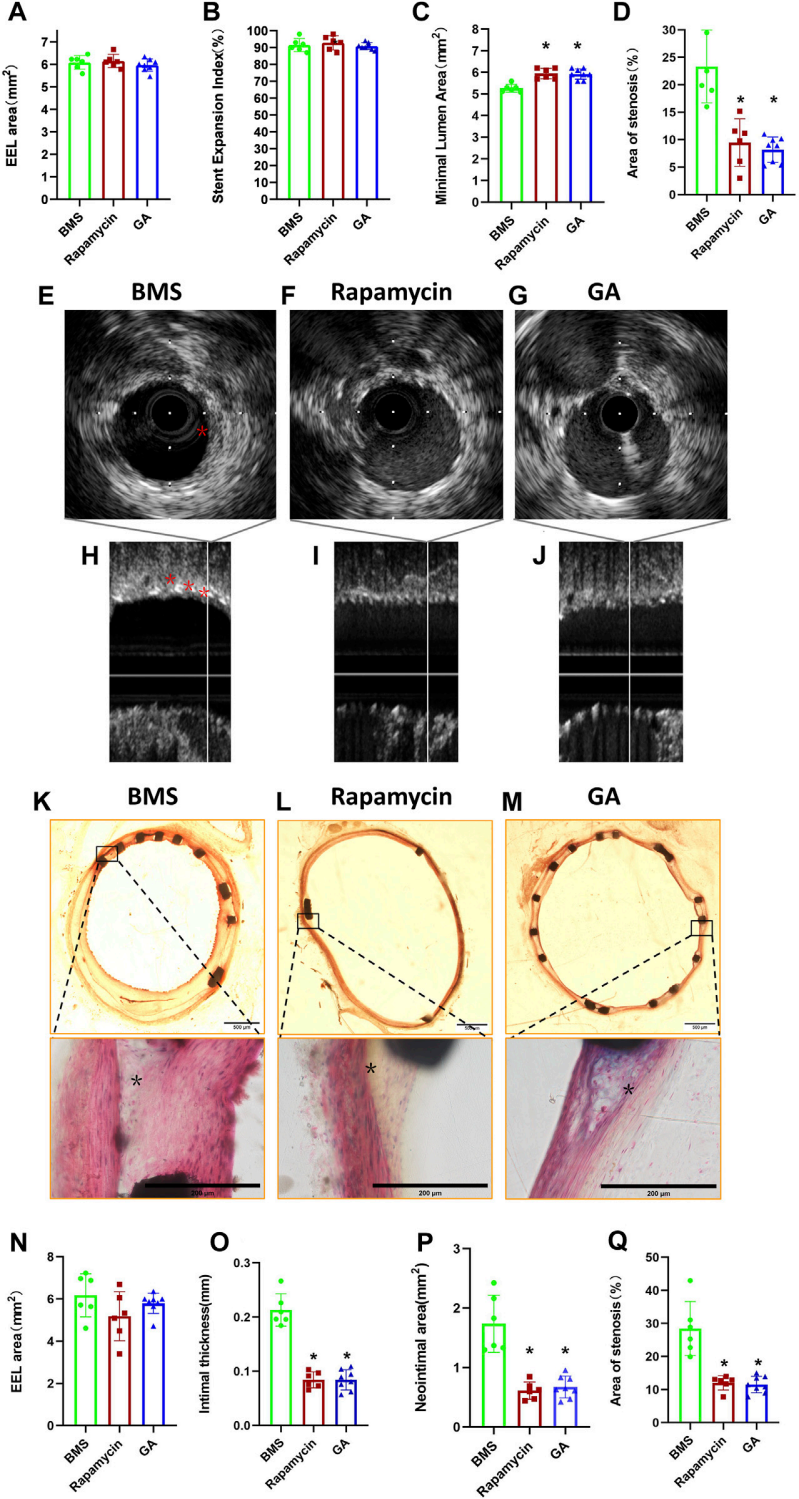
IVUS and histological analyses of restenosis of BMSs, rapamycin-eluting stents, and GA-eluting stents post 28 days of implantation. **(A)** EEL area, **(B)** stent expansion index, **(C)** minimum lumen area, and **(D)** stenosis area for BMSs, rapamycin-eluting stents, and GA-eluting stents evaluated by IVUS. *n* = 6–8. ∗ p<0.05 *vs.* BMSs. Cross-sectional images and longitudinal views of IVUS images of the representative cases of BMSs **(E, H)**, rapamycin-eluting stents **(F, I)**, and GA-eluting stents **(J, G)** (* atherosclerotic plaques). Representative cross-sectional images of low- (×2) and high-magnification (×40) hematoxylin–eosin staining of BMSs **(K)**, rapamycin-eluting stents **(L)**, and GA-eluting stents **(M)** in atherosclerotic rabbit iliofemoral arteries ( ${}^{*}$ atherosclerotic plaques). **(N)** EEL area, **(O)** intimal thickness, **(P)** neointimal area, and **(Q)** area of stenosis assessed by histological analysis.

To clarify the extent of restenosis in different stents, we first examined iliac stents using IVUS before animal euthanasia. As shown in Figure 2, there were no significant differences in the EEL area (Figure 2A) and stent expansion index (Figure 2B) among groups (6.09 ± 0.31 mm^2^ and 91.5% ± 3.8% for the BMS group, 6.15 ± 0.29 mm^2^ and 92.8% ± 4.2% for the rapamycin group, and 5.97 ± 0.27 mm^2^ and 90.9% ± 2.0% for the GA group; p > 0.05 ). However, the results showed that the minimal lumen area of rapamycin-eluting stent and GA-eluting stent groups was significantly larger than that of the bare-metal stent group (5.95 ± 0.24 mm^2^ *vs.* 5.27 ± 0.18 mm^2^; 5.93 ± 0.24 mm^2^ *vs.* 5.27 ± 0.18 mm^2^, respectively; p < 0.05 ) (Figure 2C). In addition, the mean stenosis of rapamycin-eluting stent and GA-eluting stent groups was also significantly lesser than that of the bare-metal stent group (12.0% ± 2.2% *vs.* 28.5% ± 8.2%; 11.5% ± 2.5% *vs.* 28.5% ± 8.2%, respectively; p<0.05 ) (Figure 2D). There were no differences in the minimal lumen area or mean stenosis between the rapamycin- and GA-eluting stent groups.

Consistent with the IVUS results, histological observations showed that there were no significant differences in the EEL area (Figure 2N) among groups (6.51 ± 0.47 mm^2^, 6.39 ± 0.27 mm^2^, and 6.22 ± 0.35 mm^2^, respectively; p > 0.05 ). Both GA- and rapamycin-eluting stents reduced neointimal thickness compared with bare-metal stents (Figure 2O; p < 0.05 ). Most importantly, compared with the bare-metal stent group, there was less lumen stenosis of rapamycin-eluting stent (12.02% ± 2.2% *vs.* 28.5% ± 8.2%, respectively; p<0.05 ) and GA-eluting stent groups (11.5% ± 2.5% *vs.* 28.5% ± 8.2%, respectively; p < 0.05 ) (Figure 2Q). There were no significant differences between the rapamycin- and GA-eluting stent groups. Moreover, the neointimal area was greater in the GA-eluting stent and rapamycin-eluting stent groups than in the bare-metal stent group (1.87 ± 0.59 mm^2^ *vs.* 0.51 ± 0.09 mm^2^; 1.87 ± 0.59 mm^2^ *vs.* 0.53 ± 0.08 mm^2^, respectively; p < 0.05 ) (Figure 2P).

### 3.3 Endothelial coverage by *en face* SEM after 28 days

**FIGURE 3:**
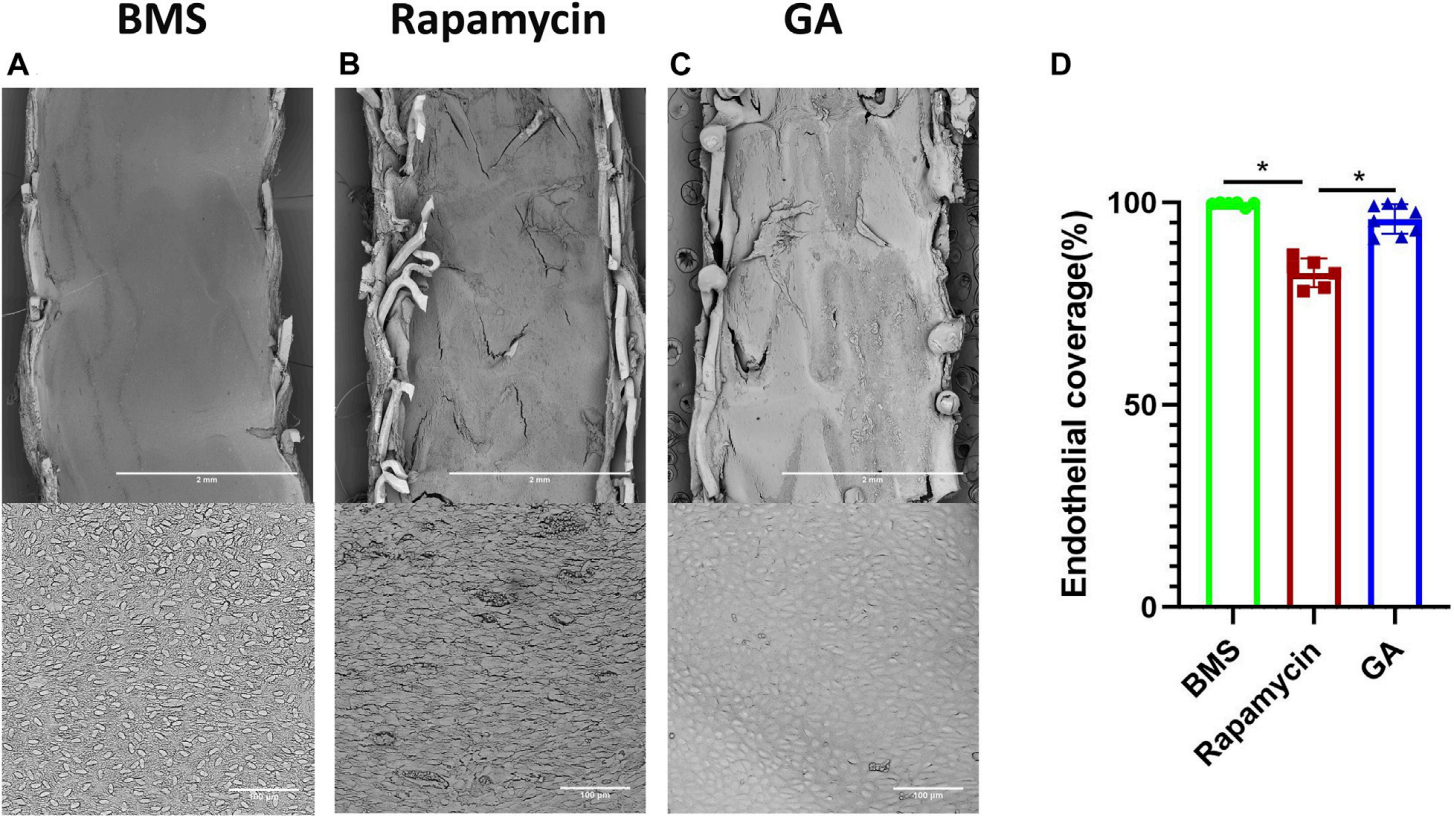
SEM and quantitative analysis of 28-day rabbit iliac artery stent implants. **(A–C)** Representative SEM images of 28-day BMSs and comparator DESs implanted in the atherosclerotic rabbit iliofemoral artery (at ×15 magnification), whereas the corresponding higher-power images (×600 magnification) from each stent are shown underneath. **(D)** Quantitative analysis of endothelial coverage of different stents. *n* = 6–8. ∗ p<0.05 .

To verify the endothelialization of each stent type, we examined the samples using SEM. As shown in Figure 3, the representative SEM images of the implanted stents after 28 days were obtained. The estimated endothelial coverage of the luminal surface was 96% for GA-eluting stents, 99% for BMSs, and 83% for rapamycin-eluting stents, indicating that a few rapamycin-eluting stents showed evidence of delayed healing, exhibiting occasionally uncovered struts ( p < 0.05 ). The GA-eluting stents showed good healing, which was comparable to that of BMSs ( p > 0.05 ).

### 3.4 Expression of eNOS in BMSs and comparator DESs

**FIGURE 4:**
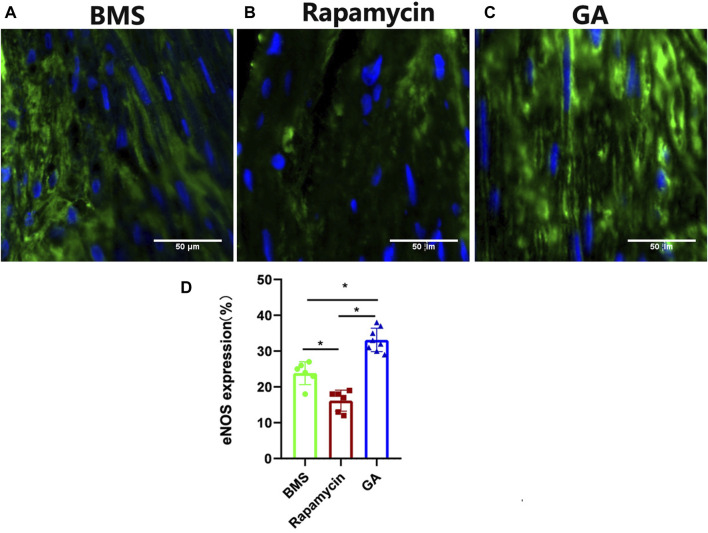
Representative images of eNOS immunofluorescence staining of 28-day stents implanted in the iliofemoral arteries of atherosclerotic rabbits. **(A)** BMSs, **(B)** rapamycin-eluting stents, and **(C)** GA-eluting stents. Images of eNOS showing reduced staining in comparator DESs relative to BMSs (green channel, eNOS; blue channel, nuclear counterstain magnification ×200). **(D)** Statistical analysis of eNOS expression in different stents. *n* = 6–8. ∗ p<0.05 .

To confirm the endothelialization of different groups, and based on our previous work showing that GA could restore eNOS expression, we tested eNOS expression in the neointima of different stents. As shown in Figure 4, eNOS expression was significantly lower in the rapamycin-eluting stent than in BMSs and GA-eluting stents (BMSs, 24% ± 3%; rapamycin-eluting stents, 16% ± 3%; and GA-eluting stents, 33% ± 3%; p < 0.05 ) (Figure 4D). In addition, eNOS expression was higher in the GA-eluting stent group than in the bare-metal stent group ( p < 0.05 ). These results indicated that GA could restore or promote the healing ability of endothelial cells after stenting.

## 4 Discussion

This study investigated the efficacy and safety of a novel drug-eluting stent in a rabbit model of atherosclerosis. The results showed that GA-eluting stents were more attractive and beneficial for endothelial cells, promoting re-endothelialization in the injured artery. Moreover, this novel stent has a similar effect in inhibiting intimal hyperplasia to the rapamycin-eluting stent, which has been broadly applied in the clinic.

Coronary artery stents have been developed for over 40 years, ranging from bare-metal stents to DESs designed to inhibit smooth muscle cell proliferation and intimal hyperplasia. Currently, DESs are the most popular stents used in PCI. Consequently, restenosis, mainly caused by intimal hyperplasia, reduced from 20%–30% in the BMS era to approximately 5%–10% in the DES era (Waksman and Steinvil, 2016; Shlofmitz et al., 2019). Bioabsorbable stents have recently emerged as a novel advancement in this area; however, negative results have been reported in clinical trials (Jabara et al., 2009; Hoare et al., 2019).

The eluting drugs were developed from paclitaxel and sirolimus (rapamycin) for first-generation DESs to zotarolimus and everolimus for second-generation DESs. Paclitaxel is a diterpenoid derivative that exerts an antineoplastic effect by interfering with microtubule activity (Zhu and Chen, 2019). It prevents the migration and proliferation of vascular smooth muscle cells stimulated by growth factors, thereby preventing neointimal formation (Sollott et al., 1995). Sirolimus and everolimus are immunosuppressants that are used post-transplantation to prevent organ transplant rejection; they both inhibit mammalian rapamycin, thereby blocking protein synthesis and cell cycle progression (Granata et al., 2016). Although the drugs eluting on the scaffold differ in structure and target proteins, they generally have the same effect of inhibiting cell proliferation and a relative lack of cell specificity.

Although intimal hyperplasia is the most crucial pathological process after stenting, re-endothelialization is a critical protective process against thrombosis in stents. However, few studies have investigated the role of endothelialization after PCI, and eluting medicine that focuses on promoting endothelialization has also been omitted. Animal and human studies have indicated that inflammation is pivotal in linking vascular injury to neointimal growth or restenosis. Anti-inflammatory therapy provides an alternative strategy for inhibiting intimal hyperplasia after stenting. GA is a pentacyclic triterpenoid glycoside that occurs naturally in substantial amounts in licorice root extract (Li et al., 2019). GA, an inhibitor of HMGB1, reduces endothelium-dependent relaxation impairment by upregulating eNOS expression in an animal model of diabetes (Zhu et al., 2020a; Zhou et al., 2021), and it attenuates neointimal formation in a rat model of iliac artery balloon injury (Zhu et al., 2020b). A previous study reported that the introduction of NO into rapamycin-eluting stents alleviated incomplete re-endothelialization (Chen et al., 2022). In this study, GA was applied to the eluting stent to inhibit intimal hyperplasia and promote re-endothelialization. A good balance between intimal hyperplasia and re-endothelialization was achieved.

Researchers are exploring new treatment options that inhibit neointimal formation while promoting stent endothelialization. Recent research studies have focused on developing polymer-free stents to prevent inflammatory responses to polymers (Worthley et al., 2017). Applying anti-CD34 on the surface of DESs promotes rapid re-endothelialization by capturing circulating endothelial progenitor cells (Nakazawa et al., 2010). Although this stent has a more rapid endothelial coverage, it has a higher risk of intimal hyperplasia. The SORT OUT X trial results with a 12-month follow-up showed that the CD34 antibody-covered sirolimus-eluting stent had a higher incidence of target lesion revascularization than the sirolimus-eluting stent (Jakobsen et al., 2021). In patients with acute coronary syndrome, there was no significant difference in stent endothelial coverage between CD34 antibody-covered sirolimus-eluting stents and everolimus-eluting stents at 60 days, as analyzed via optical coherence tomography (Jaguszewski et al., 2017). In contrast, CD31-mimetic stents preferentially promote the adherence of endothelial cells rather than smooth muscle cells or blood components (Diaz-Rodriguez et al., 2021). Stents with an endothelial-mimetic coating significantly inhibit acute thrombosis and accelerate re-endothelialization (Zhang et al., 2022). Even biodegradable vascular grafts that are 3D-printed and laden with dipyridamole have been created to achieve rapid re-endothelialization (Domínguez-Robles et al., 2021).

The novel GA-coated stent used in this study inhibited intimal hyperplasia and promoted re-endothelialization. Based on previous studies, the underlying mechanism could be attributed to HMGB1 inhibition and the promotion effect. Additionally, endothelial coverage was greater in the GA-eluting stents than in the rapamycin-eluting stents.

### 4.1 Limitations

This study had some limitations. First, although accelerated re-endothelialization was observed in the new DES, the process was not monitored at different time points. Hence, we could not record information on when the new DES completed re-endothelialization. Second, the sample size was small. Finally, although this study confirmed the effect of the novel stents, the specific mechanism remains unclear, and further studies are needed.

## Data Availability

The original contributions presented in the study are included in the article/Supplementary Material; further inquiries can be directed to the corresponding author.
