# Supplementary material for: A novel glycyrrhizin acid-coated stent reduces neointimal formation in a rabbit iliac artery model
Source: Front Pharmacol. 2023 May 17;14:1159779. doi: 10.3389/fphar.2023.1159779 (PMC10229815; doi:10.3389/fphar.2023.1159779)
Supplement: Supplementary file 1 [file Table2.DOCX]

Supplementary Material

A novel glycyrrhizin acid-coated stent reduces neointima formation in a rabbit iliac artery model

**Shuai Teng,Zhaowei Zhu, Yang Li, Xinqun Hu, Zhenfei Fang, Zhenjiang Liu*, Shenghua Zhou**

*** Correspondence:** Zhenjiang Liu, MD: zhenjliu@csu.edu.cn


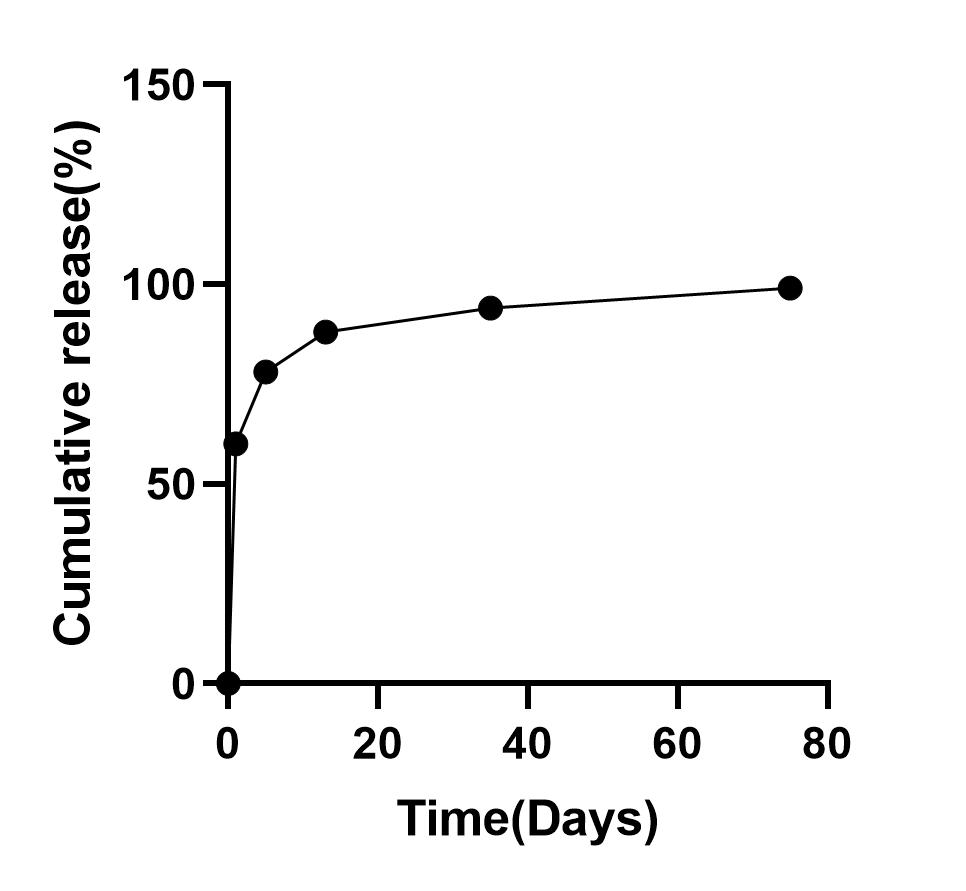


Supplementary Figure 1. GA elution kinetics. In vitro elution of GA (cumulative amount of drug released measured by UV spectroscopy).
